# Routine follow-up radiographs for distal radius fractures are seldom clinically substantiated

**DOI:** 10.1007/s00402-017-2743-6

**Published:** 2017-07-22

**Authors:** N. L. Weil, M. El Moumni, S. M. Rubinstein, P. Krijnen, M. F. Termaat, I. B. Schipper

**Affiliations:** 1 Department of Surgery, Leiden University Medical Center, Heelkunde, K6-R, P.O. Box 9600, 2300 RC Leiden, The Netherlands; 2 Department of Surgery, University Medical Center Groningen, Groningen, The Netherlands; 3 Department of Health Sciences and the EMGO Institute, VU Amsterdam, Amsterdam, The Netherlands

**Keywords:** Distal radius fracture(s), Wrist fracture(s), (Routine) radiography, Radiographs, Imaging

## Abstract

**Introduction:**

The value of routine radiographs during follow-up after distal radius fractures is unclear. The aim of this study was to evaluate whether routine radiographs performed during the follow-up period in patients with a distal radius fracture influenced clinical decision making.

**Methods:**

This retrospective cohort study included patients aged ≥18 years who were treated for a distal radius fracture at four hospitals in The Netherlands in 2012. Demographic and clinical and radiographic characteristics were collected from medical records.

**Results:**

1042 patients were included. In 121 (14%) of the 841 radiographs, a clinical indication was reported. Treatment was affected by 22 (2.6%) radiographs, including 11 (1.5%) radiographs that were categorized as routine, 9 (1.2%) of which led to prolonged cast immobilization and 2 (0.2%) to surgery for conservatively treated patients.

**Conclusion:**

Although it is common practice to take radiographs after distal radius fractures, the study results indicate that routine radiographs seldom affect treatment. This finding should be weighed against the high health care costs associated with these fractures. We hope that the results of our study will trigger the awareness among surgeons that in the current practice, many radiographs are taken on routine without influencing clinical decision making and can probably be omitted.

**Level of evidence:**

Level III.

## Introduction

Distal radius fractures are a common and costly health care problem. The incidence of this fracture is about 70–160 per 100,000 persons per year and accounts for approximately 18% of all fractures [1–3]. Routine radiography during follow-up for fractures is known to contribute to rising health care costs [4]. Health care costs have increased significantly over time, and the cost-effectiveness of diagnostic imaging has become an increasingly important consideration; nonetheless, routine radiographs remain a common practice during outpatient clinical visits by patients with a distal radius fracture [5–7].

The value of these routine radiographs is currently under discussion. Several studies that investigated the value of radiographs obtained at the first post-operative visit and post-splinting radiographs have demonstrated that radiographs without a clear clinical indication do not lead to changes in treatment strategy; however, these radiographs contribute to increased radiation exposure and greater health care costs [4, 8–12]. This discussion also applies to the use of routine radiographs during follow-up (of the fracture healing process). In general, the higher the level of expertise of the treating physician, the lower the number of control radiographs will be. Though, expert physicians cannot answer the question whether control radiographs during follow-up can be abolished completely. This is shown by Bohl et al. A survey among orthopaedic surgeons showed a large variability in the number of routine radiographs during the follow-up after surgical distal radius repair and they suggested to conduct an analysis of surgeons actual medical records [13].

Due to ageing populations, the incidence of distal radius fractures is expected to increase substantially in coming decades. It is, therefore, worthwhile to establish the clinical value of routine radiographs for monitoring fracture healing and delivering high-quality care. The aim of this study was to evaluate whether routine radiographs performed during the follow-up period in patients with a distal radius fracture influenced clinical decision making, with the hypothesis that routine radiographs in the majority of cases do not lead to changes in treatment strategy.

## Methods

### Patients

Consecutive patients from two academic hospitals and two large teaching hospitals in The Netherlands (all of which are level I trauma centres) were retrospectively analysed. Patients who were aged 18 years or older with a distal radius fracture that occurred between 1 January 2012 and 31 December 2012 were eligible for inclusion. The exclusion criteria were an absence of follow-up data, pathologic fractures, open fractures, and one or more simultaneous fractures of the extremities.

### Study procedure

A case record form was developed to extract the following data from medical records: baseline patient characteristics (age and gender); fracture type according to the Arbeitsgemeinschaft für Osteosynthesefragen/Orthopaedic Trauma Association (AO/OTA) classification [14]; treatment strategies (conservative treatment or operative treatment); radiograph dates, numbers (a series of two radiographs, i.e., AP and lateral, were recorded as one radiographic intervention), and indications (i.e. pain, new trauma, decreased range of motion (ROM), patient anxiety, etc.); and any changes in the management of fractures following radiography (conservative to operative treatment, prolonged cast immobilization, and removal of osteosynthesis material (OSM), i.e., any kind of therapy change mentioned in the medical chart was collected and stored in the case record forms). Fracture type was classified based on radiographs taken in the emergency department or during the first consultation visit (i.e., when the patient was first treated in a different emergency department).

A radiograph was designated as ‘clinically indicated’ when a clinical indication (i.e., pain, new trauma, decreased ROM, etc.) was found in the medical chart. A radiograph was designated as ‘routine’ if no clinical indication could be found in the medical chart. Furthermore, a distinction was made between radiographs that were obtained (1) during the first 3 weeks after the trauma (i.e., during the treatment period, when operations are likely to be performed) and (2) after this period (i.e., during the follow-up period).

### Statistical analysis

Descriptive statistics are reported for baseline, fracture, and radiographic characteristics. Statistics are reported for the overall group and separately for patients who received conservative treatment and patients who received operative treatment.

## Results

Among the 1375 identified patients, 333 did not satisfy the inclusion criteria; thus, 1042 patients remained for analysis. The study group consisted of 755 (72%) females and 287 (28%) males; the included patients had a mean age of 58.5 years [standard deviation (SD) 19.6 years].

In total, 804 (77%) patients received conservative treatment, and 238 (23%) patients received operative treatment. Baseline characteristics are presented in Table 1.

**Table 1:** Baseline characteristics

	Total cohort (*n* = 1042)	Conservative treatment (*n* = 804)	Operative treatment (*n* = 238)
Gender			
Male	287 (28%)	222 (28%)	65 (27%)
Female	755 (72%)	582 (72%)	173 (73%)
Age			
Mean (SD)	58.5 (19.6)	59.0 (20.3)	56.9 (17.1)
18–39 years	202 (20%)	156 (19%)	46 (19%)
40–64 years	421 (40%)	314 (39%)	107 (45%)
≥65 years	419 (40%)	334 (42%)	85 (36%)
Fracture type			
AO 23A	467 (45%)	414 (51%)	53 (22%)
AO 23B	321 (31%)	271 (34%)	50 (21%)
AO 23C	254 (24%)	119 (15%)	135 (57%)

**Fig. 1:**
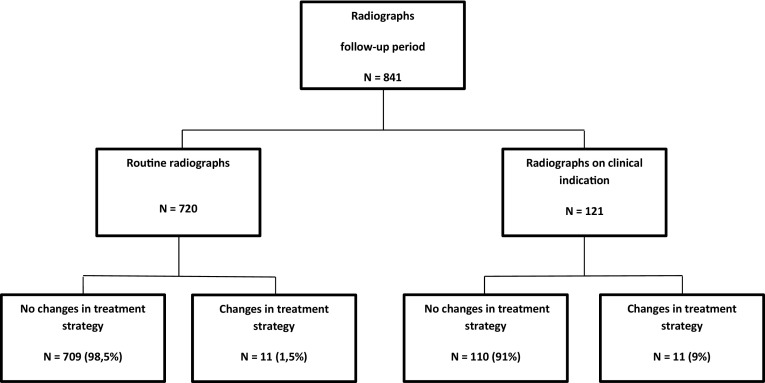
Flow chart

Table 2 provides details regarding the use of radiographs and the influence of radiographs on treatment strategy. Overall, 1956 radiographs were obtained (mean 1.88, SD 1.43). During the treatment period, 1115 radiographs were acquired (mean 1.07, SD 0.83). During the follow-up period, 841 radiographs were acquired (mean 0.81, SD 0.99). In total, 720 (86%) of the radiographs obtained during the follow-up period were categorized as routine radiographs, and in 121 (14%) radiographs, a clear clinical indication was reported. Twenty-two (2.6%) of the 841 radiographs altered treatment strategy, including 11 (1.5%) radiographs categorized as routine, 9 (1.2%) of which led to prolonged cast immobilization, and 2 (0.2%) of which led to surgery for conservatively treated patients. Figure 1 provides a flow chart regarding the radiographs and the influence of radiographs on treatment strategy during the follow-up period.

**Table 2:** Radiographic follow-up

	Total	Conservative treatment	Operative treatment
Treatment period^a^			
No. of radiographs (mean, SD)	1115 (1.07, 0.83)	754 (0.94, 0.82)	361 (1.52, 0.69)
Follow-up period^a^			
Total no. of radiographs (mean, SD)	841 (0.81, 0.99)	464 (0.58, 0.77)	377 (1.58, 1.21)
No. of routine radiographs (mean, SD)	720 (0.69, 0.86)	406 (0.50, 0.69)	314 (1.32, 1.06)
No. of radiographs on clinical indication (mean, SD)	121 (0.12, 0.40)	58 (0.07, 0.30)	63 (0.26, 0.60)
Total no. of changes in treatment strategy (%)	22 (2.6%)	12 (2.7%)	10 (2.4%)
No. of changes in treatment strategy after a routine radiograph (%)	11 (1.5%)	9 (2.2%)	2 (0.06%)

^a^Treatment period: the first 3 weeks after trauma. Follow-up period: the time after the first 3 weeks

In the conservative treated patients, 406 (87.5%) of the 464 obtained radiographs during the follow-up period were categorized as routine. Twelve (2.7%) of the 464 radiographs altered treatment strategy, including nine (2.2%) categorized as routine. In the operative-treated patients, 314 (83%) of the 377 obtained radiographs were categorized as routine. Ten (2.4%) of the 377 radiographs altered treatment strategy, including 2 (0.06%) categorized as routine.

## Discussion

The impact of routine radiographs during the follow-up of distal radius fractures on clinical decision making was assessed in a large cohort of 1042 consecutive patients.

The study results demonstrate that the vast majority of the radiographs obtained were routine and did not lead to changes in treatment strategies; in particular, only 11 (1.5%) of these routine radiographs led to an alteration in treatment strategy. Moreover, it was questionable whether these 11 radiographs were routine or were acquired due to a clinical indication that was present but not documented. Thus, we feel that it is safe to conclude that changes in treatment strategy are rarely based on routinely taken radiographs.

These findings should be considered in the context of increasing health care costs and unnecessary radiation exposure.

Our results are consistent with the findings of prior studies. Chaudhry et al. [8] demonstrated that serial radiographs in acute settings do not alter fracture management in minimally displaced fractures. In addition, Eastley et al. [9] demonstrated that for extra-articular distal radius fractures, the late displacement would not be missed if routine radiographs are removed from the guidelines. Furthermore, Huffaker et al. [15] demonstrated for operatively treated AO/OTA-type 23A fractures that 94% of the radiographs that were obtained post-operatively did not influence clinical decision making. Stone et al. [11] showed a change in treatment strategy in only three (1%) patients on the 2-week post-op radiograph. These three patients all had suffered a new trauma and would been identified clinically if radiographs were not standard at the first post-operative visit. Johnson et al. showed that an average number of 3.8 radiographs were taken per patient, while a single follow-up radiograph may been sufficient to identify complications. They concluded that their results suggest an opportunity to reduce post-operative radiographs [12]. The clinical efficiency of radiographs has been investigated for other types of fractures. These studies concluded that routine radiographs did not significantly influence clinical decision making, but did increase health care costs [4, 16, 17].

In contrast with the above-mentioned studies, this study explored the use of routine radiographs in a large cohort of patients with distal radius fractures. All fracture types (intra-articular and extra-articular fractures, displaced and undisplaced) and treatment strategies (operatively and conservatively treated patients) were included. Thus, this large cohort is an adequate representation of daily practice and may be regarded as broadly generalizable.

However, this study has certain limitations. Due to the retrospective study design, clinically relevant information that may affect fracture healing (such as smoking habits [18]) could not be retrieved from medical records for many patients. Perhaps, most importantly, clinical indications were not always documented; this issue could potentially have resulted in underestimation of the number of radiographs performed with a clinical indication. Despite this probable underestimation, the actual number of routine radiographs will still be gigantic. Therefore, these findings must be replicated in a prospective study. If the results obtained in this study are confirmed, routine radiographs may be avoided to reduce both health care expenditures and unnecessary radiation exposure. Bohl et al. [13] showed that 90% of the surgeons at this point think that it is not acceptable to reduce radiographs to save costs. Our results will hopefully trigger the awareness among surgeons that in the current practice, most radiographs are taken without influencing clinical decision making and can probably be omitted without compromising the quality of care and at the same time can save costs. Our study is the first step towards protocols with radiographs only on clinical indication; therefore, our results should be repeated in a randomized controlled trial.

## Conclusion

Although it is common practice to routinely take radiographs during follow-up for distal radius fractures, the current results suggest that these radiographs seldom influence clinical decision making. This lack of clinical relevance should be weighed against the considerations of high health care costs and unnecessary exposure to radiation.

## Abbreviations

ASA: American Society of Anesthesiologists
AO/OTA: Arbeitsgemeinschaft für Osteosynthesefragen/Orthopaedic Trauma Association
ROM: Range of motion
SD: Standard deviation
